# Neutrophilic Inflammation in the Immune Responses of Chronic Obstructive Pulmonary Disease: Lessons from Animal Models

**DOI:** 10.1155/2017/7915975

**Published:** 2017-04-23

**Authors:** Gang Huang, Xu-Chen Xu, Jie-Sen Zhou, Zhou-Yang Li, Hai-Pin Chen, Yong Wang, Wen Li, Hua-Hao Shen, Zhi-Hua Chen

**Affiliations:** ^1^Department of Respiratory and Critical Care Medicine, Second Hospital of Zhejiang University School of Medicine, Hangzhou, Zhejiang, China; ^2^State Key Lab of Respiratory Disease, Guangzhou, China

## Abstract

Chronic obstructive pulmonary disease (COPD) is a major cause of mortality worldwide, which is characterized by chronic bronchitis, destruction of small airways, and enlargement/disorganization of alveoli. It is generally accepted that the neutrophilic airway inflammation observed in the lungs of COPD patients is intrinsically linked to the tissue destruction and alveolar airspace enlargement, leading to disease progression. Animal models play an important role in studying the underlying mechanisms of COPD as they address questions involving integrated whole body responses. This review aims to summarize the current animal models of COPD, focusing on their advantages and disadvantages on immune responses and neutrophilic inflammation. Also, we propose a potential new animal model of COPD, which may mimic the most characteristics of human COPD pathogenesis, including persistent moderate-to-high levels of neutrophilic inflammation.

## 1. Introduction

Chronic obstructive pulmonary disease (COPD) is a major public health problem that is currently the fourth cause of death globally and affects about 10% of the adult population worldwide [1–3]. It is generally accepted that the neutrophilic inflammation observed in the lungs of COPD patients is intrinsically linked to the tissue destruction and alveolar airspace enlargement, leading to disease progression. Cigarette smoke injures epithelial cells and then releases “danger signals” which act as ligands for toll-like receptors (TLRs) in the epithelium. These actions contribute to the production of chemokines and cytokines, which results in an innate immunity. Products from the inflammatory cells may injure the extracellular matrix, leading to the release of TLR ligands and TLR activation, which in turn promote further inflammation and damage of lung parenchyma. Moreover, chronic cigarette smoke can induce an adaptive immune response, including CD4^+^ T cells, cytolytic CD8^+^ T cells, and B cells, leading to cellular necrosis and apoptosis, immune and complement deposition, tissue injury with airway remodeling, and emphysema [4]. Animal models act as a bridge between in vitro studies in the laboratory and studies in humans. As such, they have exerted a great impact on the investigation of many medical conditions. This review first gives an overview of the main experimental models of COPD to discuss their advantages and disadvantages in studying the neutrophilic inflammation in COPD and then try to propose a new, effective, and useful model.

## 2. Protease-Induced Emphysema Models

In 1965, Gross et al. [5] firstly proposed a model of pulmonary emphysema by instilling papain into trachea of rats. With the diagnosis of emphysema and the genetic deficiency of the protease inhibitor alpha-1-anti-trypsin [6], this animal model provided the basis for the proteinase-anti-proteinase hypothesis of human emphysema. According to this theory, various proteases, such as porcine pancreatic elastase, papain, or neutrophil elastase [7–9], have been subsequently instilled into trachea to test for the function to induce emphysema in animals. After intratracheal protease instillation, airway inflammation in the lung parenchyma, airspace enlargement, and pulmonary dysfunction (such as air trapping, alveolar destruction, and increased lung volume) have been observed [10, 11]. These features are similar to human emphysema although the speed of occurrence and development is drastically increased.

There are a number of protocols for the induction of emphysema by proteases. Authors have shown that the severity of the protease-induced emphysema is related to the dosage and frequency of the protease [12]. In a murine model, the damage caused by protease persists after 4 weeks of administration, and the application of repeated doses can result in severe cases of the disease [13–15].

Protease-induced emphysema models have a number of advantages, such as simple operation, inexpensive, and high efficiency. The characteristics similar to COPD appear quickly compared to other methods [13], such as the use of cigarette smoke. Moreover, the severity of disease depends on the dosage and frequency of the enzyme, and protease-induced emphysema is related to the epithelial and endothelial cell apoptosis, extracellular matrix degradation, and presence of oxidative stress, which make it suitable to study every phase in emphysema and to investigate neutrophilic inflammation in COPD. Other interesting aspects of this model are that it can reproduce various features of the human disease [16–18], especially the morphology of the lung parenchyma destruction, and that the induced morphological and functional changes are detectable for a long time.

Generally, it takes quite a long time for the development of COPD in human. Despite that the model reproduces many characteristics of human emphysema, this protease-induced emphysema model does not mimic a continuous low-level inflammatory process, especially the adaptive helper T cell immune responses induced by tobacco smoke, which is the most risk factor of COPD. This model neither provides the exact mechanism of alveolar destruction and the sequence of pathological events [17].

## 3. Tobacco Smoke-Induced Emphysema Models

Tobacco smoke is the most risk factor for COPD [19]. Epidemiology studies demonstrate that the incidence of COPD in smokers is higher than that in nonsmokers. Exposure to tobacco smoke can continuously induce inflammation (inflammatory cell influx and increases of cytokines and chemokines in the airway and parenchyma), mucus hypersecretion (goblet cell metaplasia), emphysema (alveolar enlargement and increased lung volume), airway remodeling (smooth muscle deposition, matrix deposition, and fibrosis), and lung dysfunction [20]. Thus, the use of tobacco smoke-induced animal models can reproduce the real process in the development of COPD, especially for investigating the pathophysiological mechanisms.

Since Huber et al. [21] have firstly described a detailed study regarding smoke-induced emphysema in animals, there are a wide variety of exposure protocols for this model. Using tobacco smoke-induced model with single, multiple, or chronic exposure regimes can have different insights into the disease pathology. Acute tobacco smoke exposure (Table 1) has been demonstrated the presence of inflammatory infiltrate in the pulmonary parenchyma (the increased number of inflammatory cells and cytokines) [22], whereas chronic exposure (Table 2) probably provides the most similar animal model of human COPD because it induces disease (emphysema, epithelial cell metaplasia, airway remodeling, and decline in lung function) with the same stimulus, rather than just inflammation [23]. At present, there are two major exposure modes, whole-body smoke exposure [24] and nose-only smoke exposure [25].

Smoke-induced animal models of emphysema can not only reproduce the pathological process in the development of COPD but also provide the opportunity to test the effect of viral or bacterial infection in the presence of emphysema development which contributes to the acute exacerbation of COPD. Meshi et al. [33] have found that in guinea pigs, chronic cigarette smoke exposure caused lesions similar to human centrilobular emphysema and that latent adenoviral infection combined with cigarette smoke exposure caused an excess increase in lung volume, air-space volume, and lung weight and a further decrease in surface-to-volume ratio compared with smoke exposure alone. In addition, tobacco smoke exposure faithfully recapitulates the predominant lung TH1 and TH17 responses that have previously been demonstrated as the characteristic of human emphysema. Thus, this model is suitable for studying COPD pathogenesis, especially for the T cell immunity.

Despite the smoke-induced animal models of emphysema are widely accepted worldwide, they still have a lot of disadvantages [23]. Firstly, the model is time- and energy-consuming (about 6 months). Secondly, there is no uniform standard for exposure method, since the dose, time, and animal strains may lead to different conclusions with same stimulator. Thirdly, the airway inflammation in this model is weak and the mucus expression is not obvious, which is not suitable to study about the mechanisms, especially for the neutrophilic inflammation in COPD [23]. The major limitation of this methodology, however, is the fact that even the COPD patients have left the smoking habit, the progression of disease still occurs. In experimental models, this phenomenon is not observed, since the end of exposure results in stable and nonprogressive emphysema [34]. Furthermore, species and strain differences must be taken into consideration when selecting an appropriate model. For instance, it appears that guinea pigs will acquire vascular alterations with smoke that are not found in standard rat models [35, 36].

## 4. Chemical Drug-Induced Airway Inflammation Models

Authors have found that various kinds of chemical drugs could induce inflammation and emphysema in pulmonary parenchyma, including lipopolysaccharides (LPS) [37], cadmium chloride, sulfur dioxide, and so on.

### 4.1. LPS-Induced Models

LPS is a major proinflammatory glycolipid component in the gram-negative bacterial cell wall. It can present as a contaminant on airborne particles and exist in cigarette smoke and air pollution. A single large dose of LPS instillation causes an inflammatory response that is combination with mucus hypersecretion and bronchoconstriction [38, 39], which mimic acute exacerbations, either given alone or given to animals also receiving cigarette smoke.

Long-term usage of bacterial LPS alone or together with short periods of exposure to cigarette smoke can induce emphysema in animals. For instance, a hamster emphysema model induced by installation of LPS twice per week for 4 weeks produced enlarged air spaces and remodeled airways with thickened walls and increased goblet cells. These changes resemble human emphysema and small airway remodeling [40].

### 4.2. Cadmium-Induced Models

Several reports have suggested that cadmium chloride (CdCl2) can reproduce experimental emphysema in animals [41, 42]. A few days after CdCl2 instillation, it causes an increase in vascular permeability with enhanced migration of polymorphonuclear leucocytes (PMN) and macrophages. Polymorphonuclear leucocyte recruitment plays an important role in enhancing inflammatory process and impairing the oxidant–anti-oxidant balance. Moreover, proinflammatory cytokines, such as matrix metalloprotease (MMP), are also related to cadmium-induced emphysema [43–45]. Kirschvink et al. [46] have shown that cadmium-induced emphysema in rats is dependent of pulmonary inflammation as well as of MMP production, as the increased MMP-2 and MMP-9 production contributes to the development of emphysema.

### 4.3. Sulfur Dioxide-Induced Models

As an irritant gas, sulfur dioxide can melt in water and become H_2_SO_3_ after intratracheal inhalation. H_2_SO_3_ can damage airway epithelium, and chronic exposure of rats to high concentrations of SO_2_ gas causes lesions similar to those seen in human chronic bronchitis. Shore et al. [47] have found that rats exposed to 250 ppm SO_2_ gas, 5 hours a day, 5 days a week, for a period of 4 weeks caused a small but significant increase in pulmonary resistance (RL) and a decrease in dynamic compliance (Cdyn). Kodavanti et al. [48] have shown that SO_2_-induced model could produce emphysema and bronchitis similar to human COPD through pulmonary function test.

COPD is a chronic pathological process, and the accumulation of neutrophils and macrophages in trachea can contribute to airway remodeling, which causes ventilatory disorder gradually [49]. Chemical drug-induced airway inflammation model can only mimic lesions of airway and pulmonary parenchyma in COPD rather than reproduce the chronic pathological process, and these models are not recommended for investigation of COPD pathogenesis. However, the airway inflammation in this model is strong enough and the observed inflammatory and pathologic changes mimic those observed in human subjects with COPD, suggesting that this murine model could be applicable to dissect the role of inflammation in the pathogenesis of these disease conditions.

## 5. Genetic Models

Epidemiology studies have found that not all smokers are equally susceptible to toxicants (toxic particles and gases, mostly tobacco smoke) and only a percentage of them develop the disease. Another interesting aspect of observation is that COPD shows familial aggregation, suggesting that the genetic background of the smoker is a key element for COPD development [4, 50]. According to the importance of gene, various studies produced emphysema models using either naturally occurring mouse strains or laboratory-produced animals that either overexpress or knock out particular genes (Tables 3–5). We summarized briefly herein their types, advantages, and disadvantages.

Genetically altered animals can not only allow research of the effects of a specific gene/protein on almost all different anatomic lesions of COPD but also potentially be useful in designing therapeutic agents. In order to link genetic predisposition and environmental factors, genetic models have also been used in combination with cigarette smoke exposure to mimic the natural condition of the onset of COPD [82, 83].

However, different mouse strains have a variety of genetic differences and they correspond to the phenotype of extreme monogenic individuals that probably does not adequately reproduce the most common forms of COPD, thus presenting limitations in terms of the translation of the results to the human disease [84, 85]. Moreover, genetic operation is a difficult and lengthy process, since the inactivation of a gene sometimes causes lethal effects and in some cases do not produce a phenotype due to overlapping functional gene [86, 87]. In view of this point, pulmonary cell specifically genetic alternations are highly encouraged to demonstrate the function of a specific gene in COPD pathogenesis. In addition, the studies of genetically altered animals usually focus on the different anatomic lesions in mouse lung rather than the chronic pathological process, so that these animal models may be inappropriate to study the neutrophilic inflammation in COPD.

## 6. Emphysema Models Based on Autoimmunity

Some COPD patients never have cigarette smoking, and the abnormal inflammatory response in patients does not resolve after quitting smoking. Furthermore, recent advances in our understanding of disease pathogenesis indicate that COPD patients exhibit many of the same features as patients suffering from classical autoimmune diseases. For instance, COPD is typified by familial predilections, the frequent presence of systemic abnormalities, and the persistence of intrapulmonary inflammation (and clinical progression) despite removal of the stimulant (e.g., tobacco smoke) [88]. In general, these observations suggest that in some patients, the pathogenesis of COPD may involve an autoimmune component that contributes to the enhanced and persistent inflammatory response [88]. It has been shown that the presence of lymphoid aggregates rich in T and B cells correlated with the severity of airflow obstruction in COPD. Also, in these patients, infiltrating CD8^+^ cell counts were related to the severity of emphysema, airway flow limitation, and the increased apoptotic epithelial and endothelial cells [89]. It has also been shown that the CD4^+^ INF-*γ* producing cells were related to the degree of airway obstruction [90], and that the CD4^+^ T cells from smokers with emphysema showed a Th17 profile, as they were able to secrete IL-17A [91]. According to the significant role of autoimmunity in progressive emphysema, Taraseviciene-Stewart et al. [92] have found that nude rats injected intraperitoneally with human umbilical vein endothelial cells (HUVECs) could produce an antibody against ECs (anti-EC humoral response), which subsequently leads to the influx of CD4 lymphocytes into the lung, apoptosis of alveolar septal cells, activation of MMPs, and eventual emphysema. In 2007, these authors also used CSE (cigarette smoke extract) intraperitoneal injection instead of xenogeneic endothelial cells to induce emphysema, and they hypothesized that CSE could act as an antigen triggering an immune response as well as oxidative stress that induced emphysema [93]. These models can reproduce emphysema, however, they use xenogeneic cells and external antigen, which cannot mimic the real pathogenesis regarding homologous autoimmunity in COPD patients. Moreover, Lee et al. have shown that the lung extracellular matrix proteins elastin, collagen, and decorin can be auto-antigen that induces the occurrence and development of COPD [94, 95]. This exciting new breakthrough may open new avenues for developing effective animal models of COPD.

## 7. Conclusion and a Perspective

As summarized above, a variety numbers of animal models have been created to attempt to reproduce human COPD, but there are still some controversial and divergent aspects regarding some methodological variables in these models. There is no model that can totally mimic the whole features in human COPD at present. Also, the lack of golden standard whether the model has been built successfully makes it difficult to analyze conclusions from different models. Thus, it is important to establish a useful and effective model which can highly mimic the human COPD characteristics.

An ideal animal model of COPD should be induced by cigarette smoke or other pathogens related to human disease, with persistent moderate-to-high levels of neutrophilic airway inflammation, typical T cell immune responses, clearly evidenced mucus hyperproduction, progressed destruction of the lung parenchyma eventually leading to epithelial apoptosis and airspace enlargement, and declined lung functions, and if possible, less time and energy-consuming.

Given the fact that cigarette smoke-induced COPD could be a Th1/Th17 predominant autoimmune disease, and there should be certain self-antigens mediate such an autoimmune adaptive response, we propose here a novel emphysema model by referencing to a standard allergen-induced asthma model [96, 97]. For such a model, we should first find the effective self-antigen which is likely produced in the lungs by cigarette smoke exposure and mediates the COPD autoimmune response. With this self-antigen, we may sensitize the mice and challenge the mice to build up an adaptive immune response model, likely exhibiting a high level of neutrophilic airway inflammation and a Th1/Th17 predominant immune response. It will be more appreciated if this model could clearly display a mucus hyperproduction as in the allergen-induced asthma model. If this could be realized, then a long-time self-antigen challenge should lead to the airway remodeling and emphysema-like airspace enlargement and eventually result in declined lung function, as does by allergens in the models of asthmatic airway remodeling [98, 99].

Nevertheless, a perfect animal model should provide a wide range of information on the pathophysiology of COPD and, as a consequence, support the development of new therapeutic approaches, resulting in a better quality of life for patients. Also, it should help us to understand more and better about the underlying mechanisms in COPD pathogenesis.

## Figures and Tables

**Table 1:** Acute tobacco smoke exposure.

Treatment	Time	Response	Reference
Twice a day, 1 hour per section, 3 days	3 days	The numbers of neutrophils and the levels of proinflammatory mediators, keratinocyte chemoattractant (KC), macrophage inflammatory protein 2 (MIP-2), and interleukin 1 beta (IL-1*β*) are all increased in bronchoalveolar lavage fluid (BALF).	[26]
Twelve cigarettes a day, three times a day	5 days	Acute exposure to cigarette smoke causes oxidative stress and increases the counts of leukocytes and macrophages and the levels of several proinflammatory cytokines, such as tumor necrosis factor alpha (TNF-*α*), IL-1*β*, interleukin 6 (IL-6), and KC.	[27]
Twenty cigarettes a day, four times a day	7 days	Acute exposure to cigarette smoke increases the number of total cells, neutrophils, macrophages, and lymphocytes in BALF and increases the levels of KC and monocyte chemotactic protein 1 (MCP-1).	[28]
Five cigarettes a day	3 days	The numbers of mobilizing neutrophils and differentiating macrophages are significantly increased in BALF, and the levels of IL-1*β*, IL-6, interferon gamma (IFN-*γ*), TNF-*α*, and MCP-1 in BALF and lung are also increased.	[29]

**Table 2:** Chronic tobacco smoke exposure.

Treatment	Time	Response	Reference
Four cigarettes a day, 5 days a week	6 months	Both Th1 and Th17 cells are significantly increased, and the levels of IL-6 and IL-17 are also increased.	[30]
Twelve cigarettes a day, 5 days a week	6 months	An increase in the total number of inflammatory cells and macrophages in BALF of mice exposed to cigarette smoke. The release of IL-1*β* and TNF-*α* is increased as well.	[31]
Four times a day, 5 minutes per section, 5 days a week	4 months	Functional IL-17A protein secreted in the lung likely establishes an autocrine loop that further induces TH17 differentiation, thereby exacerbating the effect of smoke-induced TH1 and TH17 inflammation in the lungs.	[32]

**Table 3:** Natural mutant emphysema models.

Mouse and gene	Phenotypes	Reference
Beige (Bg)	Impaired alveolar septation because of its deficiency in endosome biogenesis	[51, 52]
Blotchy (Blo)	Disruption of elastic fibers	[53]
Pallid (Pa)	Progressive emphysema because of increased collagen degradation	[54]
Tight skin (Tsk +/−)	Airspace enlargement because of impaired alveolar septation. Mice also have lower serum elastase inhibitory capacity	[55]

**Table 4:** Knockout mutant emphysema models.

Mouse and gene	Phenotypes	Reference
Tissue inhibitor of metalloproteinases-3 (TIMP-3)	Progressive emphysema from two weeks old with evidence of collagen degradation and increased MMP activity	[56, 57]
Surfactant protein D (SP-D)	Progressive airspace enlargement with 3 weeks of life. Increased macrophages with activated MMPs. The gene influences the response of alveolar epithelial type II cells to the injurious events	[58, 59]
Lysosomal acid lipase (LAL)	LAL is a key enzyme in the metabolic pathway of neutral lipids. Areas of alveolar destruction because of neutrophil influx, foamy macrophages, and Clara cell hypertrophy	[60, 61]
Klotho	Klotho is an “antiageing” hormone and transmembrane protein	[62]
Integrin beta-6 (Itgb6)	Inhibition of TGF-*β* signaling causes increased expression of MMP-12 by macrophages.	[63]
Gamma retinoic acid receptor (RAR*γ*)	Airspace enlargement because of impaired alveolar septation	[64, 65]
Platelet-derived growth factor A (PDGF-A)	Impaired alveolar septa lake of tropoelastin expression and lack lung alveolar smooth muscle cells	[66]
Growth factor receptor 3 and 4(GFR 3-4)	Absence of secondary alveoli	[67]
Fibulin-5/DANCE	It is a secreted extracellular matrix protein that functions as a scaffold for elastin fiber assembly. The model is due to the interruption of elastin synthesis	[68, 69]
Elastin	Deficient formation of air sacs	[70]
Tumor-necrosis alpha-converting enzyme (TACE)	Disabled saccular structures	[71]
Adenosine deaminase	Increased adenosine levels impair alveolar septation and induce inflammation	[72, 73]
POD-1	Hypoplastic lungs	[74]

**Table 5:** Overexpression mutant emphysema models.

Mouse and gene	Phenotype	Reference
Metalloproteinase-1 (MMP-1)	Progressive airspace enlargement because of degradation of collagen type III	[75]
Placenta growth factor (PLGF)	PLGF is an erythroblast-secreted factor. Airspace enlargement because of increased alveolar epithelial cell apoptosis	[76, 77]
Interleukin-13 (IL-13)	Increased MMP and cathepsin expression leading to emphysema	[78]
Interferon gamma (IFN-*γ*)	Progressive emphysema and increased lung compliance. Increased expression of MMPs, cathepsins, and caspases	[79, 80]
Tumor necrosis factor alpha (TNF-*α*)	Nonprogressive emphysema after 1–3 months of life	[81]
